# Arthus Reaction as an Adverse Event Following Tdap Vaccination

**DOI:** 10.3390/vaccines8030385

**Published:** 2020-07-14

**Authors:** Vitali Pool, Larissa Mege, Adel Abou-Ali

**Affiliations:** 1 Sanofi Pasteur Inc., Discovery Drive, Swiftwater, PA 18370, USA; 2 Sanofi Pasteur SA, 14 Espace Henry Vallée, 69007 Lyon, France; Larissa.Mege@sanofi.com; 3 Astellas Pharma Global Development, Inc., Northbrook, IL 60062, USA; adel.abouali@gmail.com

**Keywords:** Arthus reaction, type III immune complex mediated reaction, Tdap, tetanus toxoid, reduced diphtheria toxoid and acellular pertussis vaccine adsorbed, adverse event following immunization, VAERS

## Abstract

Repeat administration of tetanus toxoid-containing vaccines has rarely been associated with Arthus phenomenon, an immune-complex reaction. In the US, since 2013, tetanus toxoid, reduced diphtheria toxoid, and acellular pertussis vaccines (Tdap) have been recommended for administration during each pregnancy. Separately, in 2019, one Tdap was approved for repeat administration in adults in the US. We aimed to describe trends in spontaneously reported Arthus reactions following Tdap in the US and to assess the risk of this phenomenon in persons receiving Tdap repeatedly. We reviewed Arthus reports in the Vaccine Adverse Events Reporting System (VAERS), 1990–2018. Reporting rates were estimated using Tdap doses distributed data. A systematic literature review was conducted in MEDLINE for any Arthus cases reported in Tdap clinical trials and observational studies published between 2000 and 2019. We found 192 Arthus reports in VAERS after any vaccine, of which 36 occurred after Tdap and none were reported during pregnancy. The Arthus reporting rate was estimated at 0.1 per million doses distributed. We identified eight published studies of Tdap administration within five years after a previous dose of tetanus toxoid-containing vaccine; no Arthus cases were reported. We conclude that Arthus reaction following Tdap is extremely rare. Increasing frequency of repeat Tdap administration in adults in the US did not result in a detectable increase in reporting rates of this phenomenon, confirming the favorable safety profile of Tdap.

## 1. Introduction

Arthus reaction, named after the French immunologist Maurice Arthus, is currently classified as a type III hypersensitivity reaction [1]. It involves the in situ formation of antigen-antibody complexes after the injection of an antigen in a person with high levels of circulating antibodies. It manifests as local vasculitis due to the deposition of IgG-based immune complexes in dermal blood vessels [2]. In severe cases, these complexes induce a local fibrinoid necrosis with ischemia-aggravating thrombosis in the tissue vessel walls.

Cases of Arthus reaction following tetanus toxoid-containing vaccines were reported in the literature primarily during the 1960s and 1970s [3]. The reactions would generally develop over 6 to 12 hours in persons in whom tetanus revaccination was performed in the presence of high levels of circulating tetanus IgG antibodies. The reactions were characterized by pain, swelling, induration, and edema, beginning several hours after immunization and usually reaching a peak at 12 to 36 hours after immunization. These symptoms were self-limiting and resolved over the course of a few days.

Early preparations of tetanus toxoid were not precisely standardized and numerous vaccine products available on the market may have had various degrees of toxoid purification. Between the 1950s and the 1980s in the US, there were approximately 15 vaccine manufacturers (including three public health laboratories) offering tetanus toxoid, tetanus toxoid adsorbed, and tetanus and diphtheria toxoid vaccines licensed by the FDA [4]. When assessing reasons for Arthus reactions, it was noted that there was a wide-spread tendency among some providers to use tetanus vaccination repeatedly for every occurrence of wound management. These providers assumed that even though tetanus toxoid is a remarkably effective immunizing agent, it cannot be completely relied upon to protect any individual patient for more than a few months after the last injection [5]. However, some investigators have been unable to confirm a consistent correlation between more severe local reactions and high antibody levels, and thus, it is likely that other factors such as variations in toxoid purity, adjuvants, dose, and host factors may also have played a role in the development of severe local reactions [6].

In several countries, tetanus toxoid, reduced diphtheria toxoid, and acellular pertussis (Tdap) vaccines are approved for repeat administration five years or more after a previous Tdap dose. In the U.S., Tdap vaccination has been recommended for women during each of their pregnancies, since October 2012, thus, allowing for boosters administered in this population at intervals shorter than five years [7]. In January 2019, one of the Tdap vaccines in the U.S. was approved for repeat administration [8]. The objective of our review was to gather information from available data sources in order to assess the risk of Arthus reaction following vaccination, with focus on the safety of the repeat Tdap administration.

## 2. Materials and Methods

### 2.1. The Vaccine Adverse Events Reporting System (VAERS)

The Vaccine Adverse Events Reporting System (VAERS) is a national vaccine safety surveillance system created in the US in 1990 and coadministered by the Centers for Disease Control and Prevention, and the Food and Drug Administration. It receives spontaneous reports of adverse events after vaccination from healthcare providers, patients, parents, vaccine manufacturers, and others [9].

The public domain version of the VAERS data is available online at https://vaers.hhs.gov/. It excludes non-U.S. reports and duplicate reports. Individual source CVS (comma-separated values) tables (VAERS DATA, VAERS Symptoms, and VAERS Vaccine) were merged to create a flat analytical dataset using SAS® software version 9.4 (SAS Institute, Cary, NC, USA). We searched for the MedDRA preferred term “Type III immune complex mediated reaction” in reports submitted to VAERS between 11 June 1990 (the start of VAERS system database) and 14 December 2018. To calculate reporting rates of Arthus reaction following Tdap vaccines, we used doses distributed data in the US from the Immunization Services Division of the CDC (public sector doses) and IQVIA’s Drug Distribution Data (private sector doses).

VAERS data are collected via a routine government-sponsored surveillance, which does not meet the definition of research; therefore, reviews of de-identified reports are not subject to institutional review board approval and informed consent requirements.

### 2.2. Literature Review

The goal of this literature review was to identify published studies of Tdap vaccines administered in adolescents and adults who received a tetanus toxoid-containing vaccine ≤5 years previously. Tetanus toxoid-containing vaccines included pediatric diphtheria and tetanus toxoids vaccine (DT), tetanus toxoid vaccine (Ttox), adult tetanus toxoid reduced diphtheria toxoid vaccine (Td), and Tdap. We searched MEDLINE database for articles published between 2000 and 2020, using the PubMed search engine, which is a free resource developed and maintained by the National Center for Biotechnology Information at the U.S. National Library of Medicine, located at the National Institutes of Health. We applied the following MeSH terms and selection criteria as the search strategy: (“diphtheria-tetanus-acellular pertussis vaccines/adverse effects” [MeSH Terms] OR “arthus reaction” [MeSH Terms] OR (“arthus” [All Fields]) AND (“2000/01/01” [PDAT]: “2020/12/31” [PDAT]) AND “humans” [MeSH Terms].

## 3. Results

### 3.1. VAERS Reports of Arthus Reaction

Of the total of 619,479 reports in VAERS we identified 192 records coded with the “Type III immune complex mediated reaction” MedDRA term. All but one had vaccination dates between 2000 and 2018. The reports mentioned 35 different vaccines administered alone or coadministered in various combinations. The most frequently reported vaccine types, i.e., mentioned in more than 10 reports, were inactivated influenza vaccine (*n* = 39), pneumococcal polysaccharide vaccine (*n* = 39), Tdap (*n* = 36), measles mumps rubella (*n* = 20), inactivated polio vaccine (*n* = 17), Td (*n* = 16), hepatitis A vaccine (*n* = 14), meningococcal conjugate vaccine (*n* = 14), and varicella vaccine (*n* = 14).

Thirty-six reports mentioning Tdap were received between 2006 and 2018 (Table S1 in Supplemental Materials). Tdap was administered alone in 22 of these cases. In 14 persons it was coadministered with other vaccines, such as inactivated influenza, hepatitis B, hepatitis A, human papilloma virus, meningococcal conjugate, measles-mumps-rubella, and pneumococcal polysaccharide vaccines. Twenty-two (61%) vaccines were females. Among the cases with known ages, eight were 11 years of age, five were between 20 and 39, seven were between 30 and 39, six were between 40 and 49, seven were between 50 and 59, and two were between 60 and 69 years of age. In 11 persons, first symptoms of the reaction occurred on the day of vaccination; in 12 it was on the next day; in five cases the onset was on day 2 after vaccination. Six cases reported the onset of symptoms three or more days after vaccination (two on day three, two on day four, one on day six and one on day seven). Most reports provided very limited information on the clinical course and laboratory workup. There were no cases of severe sequelae, such as skin necrosis. One report represented a literature case of erythema nodosum in temporal association with Tdap vaccination (VAERS captures cases and case-series of adverse events published in the literature as spontaneous reports) [10]. This was a 39-year-old female patient, who developed several areas of pruritus and swelling on her distal lower extremities within 24 h of Tdap injection. Arthus reaction was mentioned in the manuscript as one of several differential diagnoses (the final diagnosis being vaccine-associated erythema nodosum).

No reports mentioned the use of Tdap during pregnancy.

One report (VAERS ID: 277449) mentioned the receipt of a tetanus-containing vaccine less than five years before Tdap. This 52-year-old male patient, who received a Td vaccine four years previously, developed soreness at the injection site followed by eruption (plaques of coalescing red to red brown papules over deltoid region) approximately six days after Tdap vaccination. A skin tissue punch biopsy from the deltoid area showed vascular changes compatible with small vessel vasculitis, lichenoid dermatitis with superficial and deep inflammatory infiltrate, necrotic follicle, and sebaceous gland. The diagnosis of either brachial neuritis or Arthus reaction was made.

In one reported case (VAERS ID: 744119) the injection site reactions occurred at two separate sites in an 11-year-old female patient. The report provided the following description: “Within 12 hours of Tdap and meningococcal vaccine patient developed red rash on both arms. The right (Tdap) measured 14 cm by 10 cm and the left (meningococcal vaccine) measured 4 cm by 3 cm. The Arthus reaction is usually self-limited. Antihistamines and steroid creams were given. A Tdap and meningococcal vaccine booster will not be given again for at least 10 years.” Of note, the only common component in Tdap and meningococcal conjugate vaccine is diphtheria toxoid.

**Figure 1:**
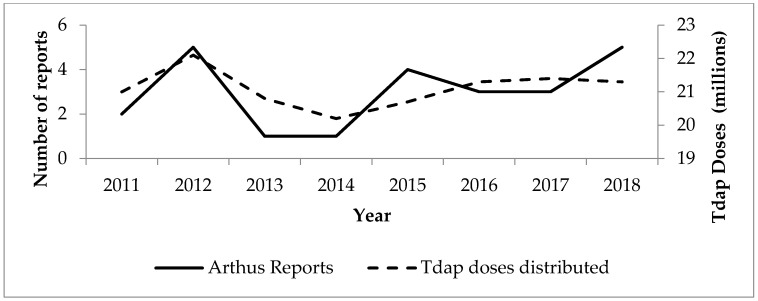
Trends in reporting of Arthus reaction following Tdap vaccines in the Vaccine Adverse Events Reporting System (VAERS) and tetanus toxoid, reduced diphtheria toxoid, and acellular pertussis vaccines (Tdap) doses distributed in the US, by year, 2011–2018.

Between 2011 and 2018, approximately 169,000,000 doses of Tdap vaccines were distributed in the US. During the same time, 24 presumed Arthus reaction cases following Tdap were reported to VAERS, for an estimated reporting rate of 0.1 per million doses distributed. Figure 1 shows trends in Arthus reaction reports after Tdap in VAERS by year, since 2011, in relation to the annual number of Tdap vaccine doses distributed.

### 3.2. Literature Review

**Figure 2:**
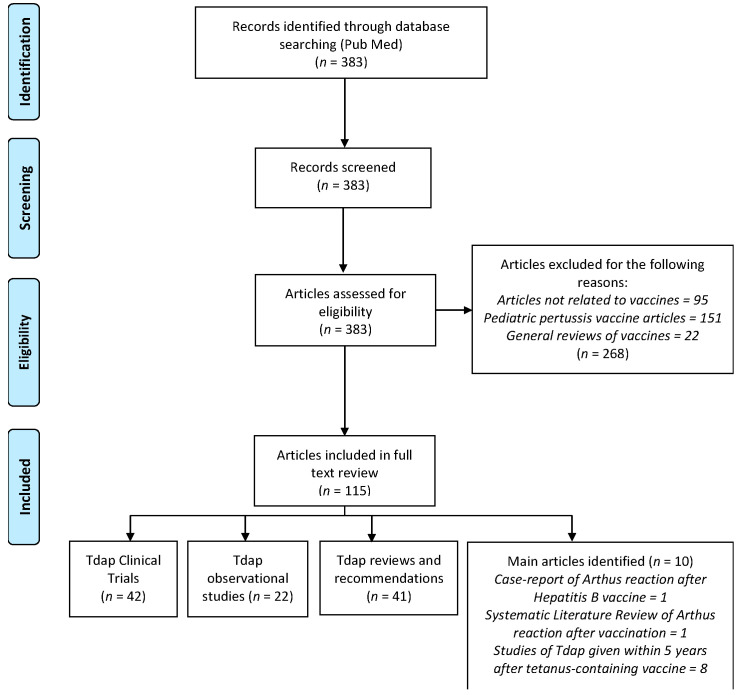
PRISMA flow diagram.

We retrieved 383 publications from MEDLINE database (Figure 2). After reviewing the titles and the abstracts, 268 articles were excluded because they did not report, or discuss, safety data for Tdap vaccine. The full text of the remaining 115 publications with information relevant to Tdap safety were reviewed in detail.

Of the 115 articles, eight manuscripts were identified as describing the safety of Tdap booster dose administered in adolescents and adults, following a dose of tetanus toxoid-containing vaccine received within five years previously. These studies are summarized in Table 1. No Arthus reaction cases have been reported by the investigators in any of these studies.

## 4. Discussion

Since the 1980s, English language literature reports and reviews of Arthus reaction following tetanus-containing vaccines have become rare [19]. This is probably due to a wider acceptance by vaccine providers of the general recommendation not to re-administer tetanus vaccines more frequently than every 10 years [20]. Other factors are undoubtedly improvements in standardization and purity of modern tetanus toxoid-containing vaccines. While two early vaccine safety reviews by the Institute of Medicine (IOM) in 1991 and 1994 provided some information on Arthus reaction after vaccination with tetanus toxoid vaccines [6,21], the most recent IOM report (2011) does not mention this adverse reaction because of its apparent lack of association with modern vaccines [22].

In this review, we found that the risk of Arthus reactions following vaccines used in the U.S. in the past two decades is extremely low. Only 36 reports were found in the VAERS database following Tdap booster. Despite increasing uptake among adults, there was no increasing trend in the reporting rate of this reaction during these years (as shown in Figure 1), including in pregnant women, in whom the Tdap coverage in 2018 reached 54% based on an Internet panel survey [23,24]. A substantial number of women receive Tdap repeatedly during subsequent pregnancies, with no apparent increase in vaccine reactogenicity [17].

Several studies identified in our literature review support the idea that Tdap vaccines do not noticeably increase the risk of injection site reactions, when administered following a recent dose of tetanus toxoid-containing vaccine (Table 1). Halperin et al. explored the safety profile of one Tdap vaccine administered at various intervals after a previous dose of DT or Td [12]. This was a nonrandomized, open-label, hypothesis-driven, single-center (province-wide) study of persons at least seven, but less than 20 years of age, who were vaccinated with Tdap at intervals ranging from 2 to 10 years after their last DT/Td vaccination. A total of 7156 were enrolled; 7001 had documented previous immunization and adverse events data were provided by 5931. The authors noted there were no reports of whole limb swelling or Arthus-like reactions by any subject in the study.

The safety of multiple doses of Tdap vaccine administered in pregnant women was evaluated in a retrospective large database study in the U.S. [17]. Adverse events following Tdap vaccination were compared between women who received a prior tetanus-containing vaccine <2 years before, 2 to 5 years before, and >5 years before. Among 29,155 pregnancies, 4812 women had a prior tetanus-containing vaccine <2 years before (in 94% of those cases, the previous vaccine was Tdap). The authors specifically mention there were no cases of anaphylaxis and Arthus reactions identified.

A comprehensive systematic literature review was conducted by Peng et al. in 2019. The authors searched MEDLINE and Web of Science and Chinese data sources (CNKI literature database and China’s AEFI surveillance system database) for any reports of Arthus reaction after vaccination, with no date or language restrictions [19]. Cases were reported after a wide variety of pediatric and adult vaccines. Most reactions reported were mild and self-limiting without treatment. The review confirmed that overall incidence of Arthus reactions was very low. No information related to Tdap vaccine was presented in the review. The authors stressed the need for the standard definition and diagnostic criteria of the reaction.

Arthus reaction following vaccination is difficult to study, because of its rarity. Currently no standard case definitions for Arthus reaction (e.g., by the Brighton Collaboration [25]) exist. It is possible that some of the few cases reported to VAERS as Arthus reaction were misdiagnosed (especially those with the onset of symptoms beyond 24 hours after vaccination), because of the lack of a standard definition and firm diagnostic criteria. These misdiagnosed cases may represent expected injection site reactions with a pathophysiological mechanism other than Type III hypersensitivity. Injection site reactions of various grades of severity are expected in a small percentage of vaccinees after both pediatric and adult tetanus toxoid-containing vaccines, and are listed in the respective prescribing information materials [8]. Like Arthus-type hypersensitivity reactions, expected injection site reactions generally appear within 48 hours after vaccination and spontaneously resolve after a few days without sequelae. The prescribing information documents for all tetanus toxoid-containing vaccines used in the U.S. provide a warning about the use of these products in persons who have experienced an Arthus-type hypersensitivity following a prior dose of a tetanus toxoid-containing vaccine. They state that such persons usually have a high level of tetanus antitoxin and that they should not receive tetanus toxoid-containing vaccines unless at least 10 years have elapsed since the last dose. Providers who suspect an Arthus reaction in adult patients receiving a tetanus toxoid- containing vaccine, including Tdap, should consider serology testing for tetanus and diphtheria antibodies. Very high concentrations measured in the patient’s blood within a few days after vaccination would support the diagnosis. Other conditions and reactions that may present similar symptoms and complaints (e.g., sterile abscess, cellulitis, SIRVA, etc.) may need to be ruled out [26].

Accurate diagnosis and confirmation of Arthus reaction is important, and, ideally, it should be a histological diagnosis with demonstration of a vasculitis. Confusing an expected injection site reaction (not Type III hypersensitivity) for Arthus reaction with the subsequent recommendation to avoid tetanus or diphtheria toxoid-containing vaccines may have negative consequences for the individual. In the case report mentioned above (VAERS ID: 744119), Arthus reaction was suspected in a teenage female after coadministration of Tdap and meningococcal conjugate vaccine. Later in life, this young female may require a second meningococcal vaccine booster to help prevent meningococcal disease through early college years, a tetanus-containing vaccine injection in case of a tetanus-prone wound injury, or a Tdap booster during a future pregnancy to help prevent her newly born infant from pertussis. Missing these opportunities to vaccinate and potentially protect against three vaccine preventable diseases will have to be balanced against the theoretical risk of the repeat Arthus episode.

Limitations of our study include those inherent to passive surveillance systems, such as VAERS, including over- or underreporting, biased reporting, inconsistency in quality and completeness of reports, and, in general, inability to assess the causality [9]. The literature review may have missed publications that were not coded with our selected MeSH terms and those that did not contain “Arthus reaction” keyword.

## 5. Conclusions

In conclusion, the risk of Arthus reaction following Tdap vaccine administration appears to be extremely low. This should provide reassurance to vaccine providers considering the increasing use of Tdap vaccines in the adult population, including in pregnant women. Future research into Arthus reaction following immunization would benefit from the development of a standard case-definition.

## Figures and Tables

**Table 1:** Studies in PubMed on the safety of Tdap vaccination in persons who received a tetanus toxoid-containing vaccine ≤5 years previously, published between 2000 and 2020.

Study Author, Publication Year (Country)	Time Since Previous Dose of Tetanus-Containing Vaccine	Number of Vaccinated Persons	Safety Findings	Study Design
David, 2006 (Canada) [11]	≥3 to <5 years versus >5 years	903	There were no significant differences in reported symptom severity between groups. Students who received Tdap ≥3 to <5 years after their last tetanus toxoid booster did not experience increased risk of severe adverse events	Prospective observational study (survey) in adolescents
Halperin, 2006 (Canada) [12]	18 months–10 years	7001	Tdap was well tolerated by all study subjects, regardless of the interval since their previous DT or Td	Nonrandomized, open-label, hypothesis-driven, single-center province-wide study in adolescents
Jackson, 2009 (USA) [13]	3–5 years	4467	No difference in risk of local reactions was identified in association with receipt of a DT-containing vaccine in the previous 5 years, or in the previous 3 years	Retrospective observational database study in adolescents and young adults
Sandora, 2009 (USA) [14]	<2, 2–5, 5–10 and >10 years	207 with completed survey	Those who received Tdap at an interval of less than 5 years after receipt of Td were significantly more likely to experience local adverse events, compared with those who reported an interval of 10 or more years	Prospective observational study (survey) in health care workers
Halperin, 2011 (USA and Canada) [15]	5 years	554	Rates of solicited and unsolicited reactions and serious adverse events were similar to those seen in other trials; “well tolerated in subjects from 16 to 69 years of age"	Phase IV, descriptive, open-label, multicenter study in adolescents and adults
Talbot, 2010 (USA) [16]	<2 years	2221	Rates of moderate & severe injection site AEs not significantly greater in those vaccinated <2 years than in ≥2 years after previous Td or Ttox	Observational study (survey) in health care workers during a pertussis outbreak
Sukumaran, 2015 (USA) [17]	<2, 2–5 and >5 years	29,155	No statistically significant differences in systemic or local reactions in <2 years group vs. 2–5 and >5 years groups; no cases of anaphylaxis, Arthus reactions or GBS	Retrospective observational database study in pregnant women
Jackson, 2018 (USA) [18]	1–5 years	61,394	Among subjects receiving a second dose of tetanus-containing vaccine <3 years after initial Tdap, receipt of Tdap vs. Td was not significantly associated with any study outcome	Retrospective observational database study in non-pregnant adults

## Supplementary Materials

The following are available online at https://www.mdpi.com/2076-393X/8/3/385/s1, Table S1: Arthus reaction reports following Tdap vaccine administration, VAERS, 1990–2018.
