# Supplementary material for: Arthus Reaction as an Adverse Event Following Tdap Vaccination
Source: Vaccines (Basel). 2020 Jul 14;8(3):385. doi: 10.3390/vaccines8030385 (PMC7563237; doi:10.3390/vaccines8030385)
Supplement: Supplementary file 1 [file vaccines-08-00385-s001.pdf]

vaccines

MDPI

Table S1. Arthus reaction reports following Tdap vaccine administration, VAERS, 1990–2018.

VAERS ID: 735537 Age: 35 Gender: F State: CA Reported on: 01/29/2018
Vaccination Date: 01/22/2018 Onset Date: 01/23/2018 Onset in days: 1
Vaccines: MMR TDAP
MedDRA PT terms: Injection site erythema Type III immune complex mediated reaction Vaccination site swelling
SYMPTOMS:
Arthus type reaction with redness and swelling in the upper arm surrounding the vaccination site. Pt. began noticing sym ptoms 1 day after vaccination. Reported she did not move are after symptoms occurred and only took Tylenol the night of 1/28/2018. No other treatment sought or completed.
Lab.Data: none
Medical History: None Reported
Other Medications: none
Prior Vaccines:

VAERS ID: 744119 Age: 11 Gender: F State: TN Reported on: 04/11/2018
Vaccination Date: 04/09/2018 Onset Date: 04/10/2018 Onset in days: 1
Vaccines: MNQ TDAP
MedDRA PT terms: Rash Rash erythematous Type III immune complex mediated reaction
SYMPTOMS:
Within 12 hours of Tdap and meningococcal vaccine patient developed red rash on both arms. The right (Tdap) measured 14cm by 10cm and the left (meningococcal vaccine) measured 4cm by 3cm. The Arthus reaction is usually self limited. Antihistamines and steroi d creams was given. A Tdap and meningococcal vaccine booster will not be given again for at least 10 years.
Lab.Data:
Medical History: None
Other Medications: None
Prior Vaccines:

VAERS ID: 745497 Age: 37 Gender: M State: MO Reported on: 04/23/2018
Vaccination Date: 04/11/2018 Onset Date: 04/13/2018 Onset in days: 2
Vaccines: TDAP
MedDRA PT terms: Feeling hot Induration Pain Type III immune complex mediated reaction
SYMPTOMS: Arthus reaction consisting of pain, warmth, and induration developed 12 hours after administration.
Lab.Data: none
Medical History: none
Other Medications: none
Prior Vaccines:

VAERS ID: 745501 Age: 46 Gender: M State: MO Reported on: 04/23/2018
Vaccination Date: 04/21/2018 Onset Date: 04/22/2018 Onset in days: 1
Vaccines: TDAP
MedDRA PT terms:
Administration site erythema Administration site induration Administration site warmth Type III immune complex mediated reaction
SYMPTOMS: Arthus reaction 10 hours after administration- erythema, warmth and induration surrounding the administration site.
Lab.Data: none
Medical History: none
Other Medications: none
Prior Vaccines:

VAERS ID: 769514 Age: 11 Gender: M State: IL Reported on: 09/06/2018
Vaccination Date: 07/31/2018 Onset Date: 07/31/2018 Onset in days: 0
Vaccines: HPV9 MNQ TDAP
MedDRA PT terms: Peripheral swelling Type III immune complex mediated reaction
SYMPTOMS: Arthus reaction in right arm started approximately 4 hours after injection. Swelling increased for 24 hours.
Lab.Data:
Medical History:
Other Medications:
Prior Vaccines:

VAERS ID: 679510 Age: 24 Gender: F State: Reported on: 01/24/2017
Vaccination Date: 11/07/2016 Onset Date: 11/10/2016 Onset in days: 3
Vaccines: TDAP
MedDRA PT terms: Type III immune complex mediated reaction

SYMPTOMS: Pt. received TDAP 11-7-16 and developed an arthus-type reaction in her right arm.
Lab.Data:
Medical History: NAPROSYN
Other Medications:
Prior Vaccines:

VAERS ID: 686280 Age: 36 Gender: F State: MO Reported on: 03/17/2017
Vaccination Date: 03/13/2017 Onset Date: 03/17/2017 Onset in days: 4
Vaccines: HEP HEPA TDAP
MedDRA PT terms: Induration Tenderness Type III immune complex mediated reaction
SYMPTOMS: Hard spot and sore to the touch, arthus-like reaction.
Lab.Data: None
Medical History: Unknown
Other Medications: No known on profile. Sore arm is left arm.
Prior Vaccines:

VAERS ID: 696041 Age: 23 Gender: F State: CA Reported on: 05/22/2017
Vaccination Date: 05/18/2017 Onset Date: 05/18/2017 Onset in days: 0
Vaccines: HPV4 MNQ TDAP
MedDRA PT terms:
Injection site erythema Injection site pain Injection site swelling Type III immune complex mediated reaction
SYMPTOMS: Arthus reaction- pain, erythema, swelling 5cmX7cm at injection site, now present for 4 days.
Lab.Data: Afebrile
Medical History: Disordered eating; No allergies
Other Medications:
Prior Vaccines:

VAERS ID: 703127 Age: 34 Gender: F State: CA Reported on: 07/10/2017
Vaccination Date: 06/28/2017 Onset Date: 07/02/2017 Onset in days: 4
Vaccines: MMR TDAP
MedDRA PT terms:
Burning sensation Cellulitis Contusion Inadequate aseptic technique in use of product Influenza like illness Peripheral swelling Pruritus Type III immune complex mediated reaction
SYMPTOMS:
Cellulitis or Arthus reaction from Tdap vaccine--Patient reported her arm was fine on 6/28 and 6/29/17, however after th at date she began experiencing increased swelling, burning, bruising and flu-like symptoms. Pt. sought care through Hosp ital ER on 7/2/17 where she stated the ER doctor informed her the site was not cleaned properly at the time of vaccinati on. She stated she was taking ibuprofen every 8 hours after receiving vaccination and was prescribed Ibuprofen 600 mg ev ery 6 hours time 3 days and Clindamycin 300 mg, 1 capsule by mouth every 6 hours x 10 days. Also stated she used cold an d warm compresses, in which the cold compress helped to relieve the itching and burning.
Lab.Data: none
Medical History: none
Other Medications: none
Prior Vaccines:

VAERS ID: 632012 Age: 11 Gender: M State: OK Reported on: 04/19/2016
Vaccination Date: 04/05/2016 Onset Date: 04/05/2016 Onset in days: 0
Vaccines: TDAP
MedDRA PT terms: Type III immune complex mediated reaction
SYMPTOMS: Arthus reaction.
Lab.Data:
Medical History: No
Other Medications:
Prior Vaccines:

VAERS ID: 633256 Age: 24 Gender: F State: VA Reported on: 04/27/2016
Vaccination Date: 04/19/2016 Onset Date: 04/21/2016 Onset in days: 2
Vaccines: TDAP
MedDRA PT terms:
Diarrhoea Headache Lymphadenitis Mass Nausea Pain Serum sickness Tenderness Type III immune complex mediated reaction
SYMPTOMS:

Lump left neck-sore to touch; headache; diarrhea; body aches; nausea; Arthus reaction. Dx: lymphadenitis, acute d/t Arth us reaction (serum sickness) diarrhea- reaction to Arthus.
Lab.Data: None
Medical History:
Other Medications:
Prior Vaccines:

VAERS ID: 567730 Age: 39 Gender: F State: Reported on: 02/04/2015
Vaccination Date: . Onset Date: . Onset in days: .
Vaccines: TDAP
MedDRA PT terms:
Erythema nodosum Nodule Peripheral swelling Pruritus Skin hyperpigmentation Skin lesion Type III immune complex mediated reaction
SYMPTOMS:
Initial report received from literature on 17 May 2014. A 39-years-old female patient, has not received any concomitant, received TDAP (batch number, route and anatomical site of administration were not reported) of unknown manufacturer on an unspecified date. It was reported that within 24 hours of receiving TDAP vaccine, the patient noted several areas of pruritus and swelling on her distal lower extremities. During the next 24 hours these areas enlarged and developed into tender red nodules. The painful nodules persisted and she sought dermatologic medical attention. Correlation of the pati ents medical history, clinical symptoms, and lesion morphology were compatible with a diagnosis of Tdap vaccine-related erythema nodosum; however, the differential diagnosis also included an Arthus like phenomenon induced by immunization an d erythema nodosum secondary to another etiology with immunization being associated by chance. Several circumstances fav ored Tdap vaccine-related erythema nodosum. Specifically, the patient had not been receiving any topical or systemic med ications. Also, she had no recent streptococcal pharyngitis or systemic conditions such as Crohn's disease, sarcoidosis, or tuberculosis. Therefore, the temporal association between her recent Tdap vaccination and the development of the ski n lesion suggested that the development of her erythema nodosum was related to her receiving Tdap vaccine. On cutaneous examination, 7 day after receiving Tdap vaccine, revealed tender erythematous nodules on her legs. There was a large, 12 by 8 cm, erythematous nodule on distal pretibial left leg. Multiple, individual and grouped, nodules were present on he r right leg; they were located on the distal lateral thigh (4.0 by 2.5 cm), the proximal lateral calf (6.0 by 3.5 cm), t he distal lateral calf (2.0 by 2.0 cm), and the distal lateral leg proximal to the ankle (each of 3 lesions measuring 2. 5 by 2.5 cm). Symptomatic treatment was initiated oral ibuprofen 600 mg four times daily. Since a drug induced etiology was susp
Lab.Data:
Cutaneous examination, 7 days after receiving Tdap vaccine, revealed tender erythematous nodules on her legs. There was a large, 12 by 8 cm, erythematous nodule on distal pretibial left leg. Multiple, individual and grouped, nodules were pr
Medical History: Unknown
Other Medications: Unknown
Prior Vaccines:

VAERS ID: 575268 Age: 11 Gender: M State: CO Reported on: 04/23/2015
Vaccination Date: 04/20/2015 Onset Date: 04/22/2015 Onset in days: 2
Vaccines: HPV4 TDAP
MedDRA PT terms: Injection site erythema Type III immune complex mediated reaction Vaccination site swelling
SYMPTOMS: Redness and swelling at vaccination site. Likely Arthus reaction.
Lab.Data:
Medical History: N/A
Other Medications:
Prior Vaccines:

VAERS ID: 587650 Age: 41 Gender: F State: KY Reported on: 07/29/2015
Vaccination Date: 07/26/2015 Onset Date: . Onset in days: .
Vaccines: TDAP
MedDRA PT terms: Type III immune complex mediated reaction
SYMPTOMS: Arthus reaction L shoulder.
Lab.Data:
Medical History: None known
Other Medications:
Prior Vaccines:

VAERS ID: 588936 Age: 38 Gender: M State: CA Reported on: 08/06/2015
Vaccination Date: 07/24/2015 Onset Date: 07/25/2015 Onset in days: 1
Vaccines: TDAP

MedDRA PT terms: Type III immune complex mediated reaction Vaccination site pain Vaccination site swelling
SYMPTOMS: Pain, marked swelling area of vaccine and throughout upper arm - suggestive of Arthus phenomenon.
Lab.Data: None
Medical History: No
Other Medications: None
Prior Vaccines:

VAERS ID: 596538 Age: 11 Gender: M State: CO Reported on: 09/21/2015
Vaccination Date: 09/16/2015 Onset Date: 09/17/2015 Onset in days: 1
Vaccines: HPV9 MNQ TDAP
MedDRA PT terms: Injection site reaction Type III immune complex mediated reaction
SYMPTOMS: Arthus reaction of right arm at injection site.
Lab.Data:
Medical History:
Other Medications:
Prior Vaccines:

VAERS ID: 555753 Age: 41 Gender: F State: MI Reported on: 11/26/2014
Vaccination Date: 11/15/2014 Onset Date: 11/16/2014 Onset in days: 1
Vaccines: TDAP
MedDRA PT terms: Injection site pain Pain Swelling Type III immune complex mediated reaction
SYMPTOMS:
Office visit notes from provider of 11/18/14: History: Onset: 4 days ago. It occurs intermittently and is improving. Location: left upper arm. The pain is aching. Associated symptoms include swelling. Additional information: pain just wi th movement, no med taken. Had Tdap injection in Saturday after cutting hand in can lid. Had injection around 6 pm in Sa turday – sx of pain and swelling in L arm started Sunday PM, Monday noticed pain radiated to neck – was stiff. Is feelin g much better today, sx seem to be resolving. Assessment: Arthus reaction. Reviewed symptomatic treatment measures and e mergency precautions reviewed, may take OTC ibuprofen 2 tablets three time daily. I called on 11/26/14 and was told left upper shoulder/neck area fully resolved by the weekend (22nd).
Lab.Data: None
Medical History: None: Allergies: sulfamethoxazole, trimethoprim
Other Medications: Ibuprofen
Prior Vaccines:

VAERS ID: 484933 Age: 64 Gender: F State: OH Reported on: 02/20/2013
Vaccination Date: 01/28/2013 Onset Date: 01/29/2013 Onset in days: 1
Vaccines: TDAP
MedDRA PT terms:
Asthenia Condition aggravated Diaphragmalgia Injection site pain Mobility decreased Neck pain Pyrexia Spinal pain Type I II immune complex mediated reaction
SYMPTOMS:
Initial case was received from a consumer on 08 February 2013. A 64-year-old female patient received an injection of Tdap (lot number, route, site, side and dose in series not reported) on 28 January 2013. The next m
orning on 29 January 2013, the patient woke up with aching in her neck, spine and diaphragm and could not turn her head side to side. Injection site was still very sore. The following day, she experienced low grade fever, and weakness. She visited her doctor. According to the report, the patient describes Arthus reaction per her doctor. On 08 February 2013, the date of report, the patient stated she had low grade fever and neck pain, but had good energy. The patient had a pas t medical history of arthus reaction and "tons" of allergies to unspecified: drugs, foods, pets and hay fever. She had n o illness at time of vaccination. She had received a shingles vaccine on an unspecified date four or five weeks prior to vaccination. Concomitant medications were not reported. Laboratory or diagnostic testing and treatments were not report ed. The patient's outcome was reported as not recovered (also reported as recovered, date not specified). Documents held by sender: None.
Lab.Data: Not reported.
Medical History:
The patient had a past medical history of arthus reaction and "tons" of allergies to unspecified: drugs, foods, pets and hay fever. She had no illness at time of vaccination. She had received a shingles vaccine on an unspecified date four o
Other Medications:
Prior Vaccines:

VAERS ID: 505634 Age: . Gender: F State: WI Reported on: 10/02/2013
Vaccination Date: . Onset Date: . Onset in days: .
Vaccines: TDAP

MedDRA PT terms: Injection site joint pain Injection site pain Type III immune complex mediated reaction
SYMPTOMS:
This case was reported by a pharmacist and described the occurrence of Arthus-type reaction in a female subject aged bet ween 50 and 59 years old who was vaccinated with TDAP. In 2012, "approximately two weeks ago", the subject received a dose of TDAP (unknown route, left arm). In 2012, less than one month after vaccination with TDAP, the subject experienced Arthus-type reaction, injection arm pain and shoulder pain in injection arm. The subject complained to the p harmacist of arm aching along with shoulder joint of left arm whenever her arm was used. The pharmacist asked how long a n Arthus type reaction may last. The subject advised the pharmacist that she received the TDAP vaccine approximately two weeks ago at the physician's office and that whenever she used her arm it ached along with her shoulder joint. The pharmacist thought it was her left arm where the subject was administered the vaccine. The pharmacist did not know when the subject received her last Td vaccine. According the pharmacist, the subject called the physician's office and they i nformed her that this was a possible reaction to the vaccine. At the time of reporting the events were unresolved.
Lab.Data:
Medical History:
It was unknown if the subject had any other vaccines given on the same date, any concomitant medications and any relevan t medical history. The pharmacist did not know when the subject received her last Td vaccine.
Other Medications:
Prior Vaccines:

VAERS ID: 451776 Age: 11 Gender: F State: MN Reported on: 03/09/2012
Vaccination Date: 02/08/2011 Onset Date: 02/08/2011 Onset in days: 0
Vaccines: HEPA HPV4 MNQ TDAP
MedDRA PT terms: Erythema Injection site reaction Oedema peripheral Type III immune complex mediated reaction
SYMPTOMS:
Initial report was received from a health care professional on 10 February 2011. An 11 year-old female patient with a hi story of asthma, eczema, a peanut allergy and environmental allergies had received on 08 February 2011 a first dose intr amuscular right deltoid injection of meningococcal vaccine, lot number U3518AA, a first dose intramuscular right deltoid injection of TDAP, lot number C3489AA, GARDASIL (Merck lot number, route and site of administration not reported) and Hep A (manuf acturer, lot number, route and site of administration not reported) and an unspecified amount of time later the same day , experienced a Arthus reaction of swelling and redness of the right upper arm down to the elbow. The patient was evalua ted by a physician. Treatments were not reported. Concomitant medications included ADVAIR, Albuterol, FLONASE, CLARITIN and an EPIPEN. According to the reporter the patient had a "similar reaction" after she had received an IPOL and MMR vac cine. The patient also had to be revaccinated after having a DTaP vaccine when she was five year old because it was thou ght that the vaccine had been "unstable" from being too cold. No further information was available at the time of the re port. The patient's outcome was unknown. Documents held by sender: none.
Lab.Data:
Medical History:
The patient has a history of asthma, eczema, environmental allergies and an allergy to peanuts. She had no illness at th e time of vaccination.
Other Medications: ADVAIR; Albuterol; FLONASE; CLARITIN; EPIPEN
Prior Vaccines: Adverse event NOS~Measles + Mumps + Rubella (no brand name)~UN~0.00~Patient|Adverse event NOS~Polio
----

VAERS ID: 452628 Age: 31 Gender: M State: CA Reported on: 03/29/2012
Vaccination Date: . Onset Date: . Onset in days: .
Vaccines: TDAP
MedDRA PT terms:
Injection site erythema Injection site oedema Injection site pain Injection site swelling Injection site warmth Type III immune complex mediated reaction
SYMPTOMS:
Arthus or arthus-like localized reaction around site of injection with swelling, erythema, edema, pain & heat within 12 hours after injection.
Lab.Data: Exam only
Medical History:
Other Medications:
Prior Vaccines:

VAERS ID: 453914 Age: 62 Gender: M State: NE Reported on: 04/17/2012
Vaccination Date: 03/16/2012 Onset Date: 03/18/2012 Onset in days: 2
Vaccines: TDAP
MedDRA PT terms:
Activities of daily living impaired Asthenia Fatigue Mobility decreased Muscular weakness Musculoskeletal pain Neck pain Pain Spinal pain Type III immune complex mediated reaction
SYMPTOMS:

(3/21) Generalized aching & fatigue at first then neck & shoulder pain. (3/26) Missed day of work & pain increased & sta rted having weakness in the left arm. Was seen by provider on 4-2-2012 without intense pain over left shoulder extending to cervical spine with weakness & loss of motion. Dx: Arthus reaction to Tetanus prophylaxis.
Lab.Data: Physical examination
Medical History: NKDA
Other Medications:
Prior Vaccines:

VAERS ID: 457115 Age: 44 Gender: F State: MN Reported on: 06/11/2012
Vaccination Date: 06/06/2012 Onset Date: 06/07/2012 Onset in days: 1
Vaccines: TDAP
MedDRA PT terms:
Injection site erythema Injection site nodule Injection site swelling Type III immune complex mediated reaction
SYMPTOMS:
Patient had softball size red swollen reaction at site of injection with quarter size darker red hard kast at site. KEFL EX 500 mg TID x 7 days. Arthus reaction.
Lab.Data:
Medical History: Allergies: Avocado; Latex; PERCOCET
Other Medications:
Prior Vaccines:

VAERS ID: 470361 Age: 58 Gender: M State: NC Reported on: 10/18/2012
Vaccination Date: 10/01/2012 Onset Date: 10/02/2012 Onset in days: 1
Vaccines: TDAP
MedDRA PT terms:
Alanine aminotransferase increased Anion gap Anti-cyclic citrullinated peptide antibody positive Antinuclear antibody ne gative Aspartate aminotransferase normal Basophil percentage decreased Blood 25-hydroxycholecalciferol decreased Blood a lbumin normal Blood alkaline phosphatase normal Blood bilirubin increased Blood calcium decreased Blood chloride normal Blood cholesterol Blood creatine phosphokinase normal Blood creatinine normal Blood glucose normal Blood osmolarity decr eased Blood potassium normal Blood sodium decreased Blood urea normal Blood uric acid normal C-reactive protein increase d Carbon dioxide increased Eosinophil percentage Erythema Glomerular filtration rate normal Haematocrit normal Haemoglob in normal Induration Laboratory test abnormal
SYMPTOMS:
Presented to office for evaluation on 10/3/12. MD documented a 23cm x 17cm horizontal erythematous, indurated, warm, pa inful area extending from his lateral bicep and going down just below his olecranon. He diagnosed the pt. with presumed cellulitis and administered Rocephin 1gm IM. He returned to the office on 10/4/12 for follow up with another Dr. His symptoms had worsened and he was sent to Hospital where he was admitted. He was seen by infectious disease and determin ed not to have cellulitis, but was instead diagnosed with an "Arthus-type reaction" to a Tdap vaccine.
Lab.Data:
WBC: 8.0 x10^3/uL, Date/Time: 10/03/2012 17:25, Normal Low: 3.2, Normal High: 11.8, Critical Low: Critical High: Status: Auth (Verified); WBC: 5.9 x10^3/uL, Date/Time: 10/01/2012 11:45, Normal Low: 3.2, Normal High: 11.8, Critical Low: Crit
Medical History:
Arthritis, rheumatoid/ICD-9-CM 714.0/Confirmed; Bone Spur/ICD-9-CM 726.91/Confirmed; BPH/ICD-9-CM 600.00/Con firmed; Cervical Radiculopathy at C6/ICD-9-CM 723.4/Confirmed; Chest Pain/ICD-9-CM 786.50/Confirmed; Chronic Pai
Other Medications:
Prior Vaccines:

VAERS ID: 426517 Age: 40 Gender: F State: NE Reported on: 06/30/2011
Vaccination Date: 06/28/2011 Onset Date: 06/29/2011 Onset in days: 1
Vaccines: PPV TDAP
MedDRA PT terms:
Full blood count abnormal Lymphadenopathy Lymphocyte count Neutrophil count Type III immune complex mediated reaction Va sculitis White blood cell count
SYMPTOMS:
W/i 12 hrs of getting vaccine, patient developed an arthus reaction (local vasculitis) & swollen lymph nodes. Urgent Car e treated w/KEFLEX for possible cellulitis. Dr. gave Prednisone 20 mg BID x 5 days.
Lab.Data: CBC WNL except: WBC= 14; Neutrophil: 84; ANC: 11.7; Lymphocyte = 9
Medical History: All: AMBIEN; Med. Conditions: Migraines, Insomnia, Tobacco abuse
Other Medications: EXCEDRIN Migraine; Calcium; MOTRIN
Prior Vaccines:

VAERS ID: 377323 Age: 11 Gender: F State: CA Reported on: 01/19/2010

Vaccination Date: 01/07/2010   Onset Date: 01/09/2010   Onset in days: 2
Vaccines: FLUN TDAP
MedDRA PT terms: Type III immune complex mediated reaction
SYMPTOMS: L arm arthus reaction.
Lab.Data:
Medical History: None
Other Medications:
Prior Vaccines:

VAERS ID: 338070   Age: 58 Gender: F State: ID Reported on: 01/23/2009
Vaccination Date: 11/24/2008   Onset Date: 11/25/2008   Onset in days: 1
Vaccines: TDAP
MedDRA PT terms: Cellulitis Injection site erythema Injection site warmth Type III immune complex mediated reaction
SYMPTOMS:
Received TDAP on 11/24/08 for travel to Brazil. Returned to clinic on 11/28/08 for an arthur-type reaction left deltoid , 10 × 10 cm, warm tissue than surrounding tissue, pale center, redder surrounding. 12/11/08 client saw MD on 11/28. D x with cellulitis, client received antibiotics.
Lab.Data: N/A
Medical History: None
Other Medications: CRESTOR
Prior Vaccines:

VAERS ID: 340381   Age: 11 Gender: M State: WV Reported on: 02/23/2009
Vaccination Date: 02/13/2009   Onset Date: 02/13/2009   Onset in days: 0
Vaccines: MNQ TDAP
MedDRA PT terms:
Injection site erythema Palpitations Rash erythematous Tenderness Type III immune complex mediated reaction
SYMPTOMS:
Mom states erythema in shot area started day of injection, worsened through weekend. Presented to Clinic 2/16 with large (about 10 × 11 cm) erythematous area on left upper arm, mildly tender to palpation, no fevers, no induration. Diagnosed with arthus reaction.
Lab.Data: None
Medical History:
All: INVEGA; crab meat; med; conditions: ADHD; Bipolar disorder, DDD, Mild persistent asthma; Obesity; Allergic rhinitis
Other Medications:
Prior Vaccines:

VAERS ID: 340883   Age: 53 Gender: F State: MI Reported on: 03/01/2009
Vaccination Date: 02/24/2009   Onset Date: 02/24/2009   Onset in days: 0
Vaccines: TDAP
MedDRA PT terms: Myalgia Pyrexia Type III immune complex mediated reaction
SYMPTOMS: Fever, myalgias, Arthus reaction
Lab.Data: none
Medical History: Prozac, hyper feeling
Other Medications:
Synthroid 175mcg, daily; Prinzide 20/12.5mg, daily; Aspirin 81mg, daily; Hydrocodone/acetaminophen 5/500mg, as needed; A mbien 10mg, as needed; acetaminophen 500mg, as needed.
Prior Vaccines: none~ ()~NULL~~In Patient

VAERS ID: 351443   Age: 51 Gender: M State: NY Reported on: 07/15/2009
Vaccination Date: 07/13/2009   Onset Date: 07/14/2009   Onset in days: 1
Vaccines: TDAP
MedDRA PT terms: Injection site reaction Type III immune complex mediated reaction
SYMPTOMS: Typical Arthur reaction at right deltoid injection site
Lab.Data: None
Medical History:
Other Medications:
Prior Vaccines:

VAERS ID: 324010   Age: 40 Gender: F State: CO Reported on: 09/03/2008
Vaccination Date: 08/29/2008   Onset Date: 08/30/2008   Onset in days: 1

Vaccines: HEP TDAP
MedDRA PT terms: Chills Injection site reaction Malaise Pyrexia Tremor Type III immune complex mediated reaction
SYMPTOMS:
24-48 hours post vaccination of Tdap (r delt) and Hep B (left Delt) pt became feverish, 104 deg, shakes, chills, in bed with malaise. Artus reaction in Right arm. Pt self treated R arm with "Celestial Seasons" muscle balm, which made it wor se-contained menthol.
Lab.Data: None
Medical History: None
Other Medications: None
Prior Vaccines:

VAERS ID: 277449 Age: 52 Gender: M State: TX Reported on: 04/26/2007
Vaccination Date: 04/03/2007 Onset Date: 04/09/2007 Onset in days: 6
Vaccines: TDAP
MedDRA PT terms:
Biopsy skin abnormal Blister Blood uric acid Complement factor abnormal Dysaesthesia Eosinophil count Eosinophil count i ncreased Haemoglobin normal Inflammation Injection site oedema Injection site pain Lichenoid keratosis Lymphocyte count Mean cell haemoglobin concentration Mean cell haemoglobin normal Mean cell volume abnormal Monocyte count increased Musc uloskeletal pain Neck pain Neutrophil count Paraesthesia Platelet count normal Radiculitis brachial Rash papular Red blo od cell count decreased Skin lesion Type III immune complex mediated reaction Varicella Vasculitis White blood cell coun t normal
SYMPTOMS:
Previous Td was March 2003. From HPI dated 12 Apr 07: Experienced onset of typical soreness at vaccination site followed by eruption of red raised areas. Has noticed associated dyesthesias with wearing his watch and clothing over his left a rm to his wrist. Vague pain near posterior shoulder and neck. Reports previous hx of frozen shoulder after tetanus vacci ne as a child. Left deltoid region with several raised erythematous and edematous plaques some with central papulovesicu lar change. No purpura, necrosis or crusts. Left hand no suspicious acral lesions. Apr 17: over past 5 days reports few new lesions on forearms and darker red color to older sites around shoulder. Also described episodes of 5/10 pain at sho ulder site at rest and with arm elevation. Baseline 2/10 pain. No new lesions past day or so. Has several plaques of coa lescing red to red brown papules over deltoid region with few scattered down to forearm. Some with focal superficial cru sts. Diagnosis: 1. Brachial Neuritis 2.0 Arthus Reaction. Lesions, plaque 4/09/2007, lesion Vesicle 04/09/2007. Pain 04/ 09/2007 left shoulder and neck. Edema at injection site 30-50mm 04/07/2007. Paresthesia 04/09/2007 dysesthesia.
Lab.Data:
8Apr07: Tissue punch biopsy left deltoid arm, Final diagnosis: skin left arm (deltoid area) punch biopsy: Vascular chang es compatible with small vessel vasculitis, Lichenoid dermatitis with superficial and deep inflammatory infiltrate, necr
Medical History: Denies
Other Medications: allopurinol, pravastatin
Prior Vaccines:

VAERS ID: 295715 Age: 22 Gender: F State: MI Reported on: 11/06/2007
Vaccination Date: 10/26/2007 Onset Date: 10/27/2007 Onset in days: 1
Vaccines: FLU HPV4 PPV TDAP
MedDRA PT terms: Oedema peripheral Pyrexia Type III immune complex mediated reaction
SYMPTOMS: 19cm Red Swollen area on Left Arm , fever >101.1 Arthus reaction from Tdap vaccination
Lab.Data:
Medical History: Liver Transplant for Cancer, Kidney Stones, Chronic pain,
Other Medications:
Hydrocodone/Acetaminophen, Rozerm, Restoril, Buspirone,Xopenex HFA, Allegra, Promethazine,Ortho-Novum, Ranitidine ,Keppr a, Drysol,hydroxyzine, Imodium
Prior Vaccines:

VAERS ID: 296176 Age: 34 Gender: F State: MI Reported on: 11/09/2007
Vaccination Date: 10/31/2007 Onset Date: 11/02/2007 Onset in days: 2
Vaccines: TDAP
MedDRA PT terms: Induration Type III immune complex mediated reaction
SYMPTOMS: Arthus Reaction 3-4 cm induration lateral left arm
Lab.Data: none
Medical History:
Other Medications:
Prior Vaccines:

VAERS ID: 255402 Age: 50 Gender: F State: VT Reported on: 05/12/2006

Vaccination Date: 05/09/2006 Onset Date: 05/10/2006 Onset in days: 1
Vaccines: TDAP
MedDRA PT terms:
Cellulitis Injection site hypersensitivity Injection site mass Injection site oedema Type III immune complex mediated re action
SYMPTOMS:
Noted swelling and redness with firmness at site which has increased from Quarter size to 12 × 16cm. DX'd as Arthus vs. C ellulitis. Placed on Augmentin.
Lab.Data: NONE
Medical History: Allergy Ancef sulfa.
Other Medications: Synthroid OCC, Arantadine.
Prior Vaccines:

VAERS ID: 258759 Age: 23 Gender: F State: WI Reported on: 06/23/2006
Vaccination Date: 06/20/2006 Onset Date: 06/23/2006 Onset in days: 3
Vaccines: TDAP
MedDRA PT terms: Type III immune complex mediated reaction
SYMPTOMS: ARTHUS REACTION DAY 3 AFTER INJECTION ABX
Lab.Data:
Medical History: amoxil, codeine
Other Medications:
verapamil 240 mg BID dyazide 50/25 qd propanolol 80 mg daily zomig prn
vicodin es 1-2 q 6 hours prn
Prior Vaccines:

A line listing of Type III immune complex mediated reaction reports following any Tdap (alone or co-administered with other vaccines). Public domain VAERS database, 11JUN1990-31DEC2018.
